# Effectiveness of Yijinjing on cognitive and motor functions in patients with Parkinson’s disease: study protocol for a randomized controlled trial

**DOI:** 10.3389/fneur.2024.1357777

**Published:** 2024-04-26

**Authors:** Kailiang Luo, Xinran Ma, Xueming Jin, Xinhao Liu, Yujia Li, Shujie Ma, Jun Hu

**Affiliations:** ^1^Department of Rehabilitation, The First Affiliated Hospital of Fujian Medical University, Fuzhou, Fujian, China; ^2^Department of Rehabilitation, National Regional Medical Center, Binhai Campus of the First Affiliated Hospital, Fujian Medical University, Fuzhou, Fujian, China; ^3^School of Rehabilitation Science, Shanghai University of Traditional Chinese Medicine, Shanghai, China; ^4^The Fifth Affiliated Hospital of Zhengzhou University, Zhengzhou, Henan, China; ^5^The Second Rehabilitation Hospital of Shanghai, Shanghai, China

**Keywords:** Parkinson’s disease, Yijinjing exercise, motor function, cognitive function, aging

## Abstract

**Background:**

Parkinson’s disease (PD) is a common neurodegenerative disorder that affects motor and non-motor functions, significantly reducing patients’ quality of life. No effective drug-based treatments are known to solve this problem. Non-drug therapies such as Yijinjing exercise have shown potential in improving cognitive and motor functions in PD patients. However, solid evidence must still be provided to support their clinical efficacy. This study aims to evaluate the clinical efficacy of Yijinjing exercise interventions in PD patients and explore the underlying mechanisms between the cognitive and motor functions in PD.

**Methods:**

This is a single-center randomized controlled trial in which 96 eligible PD patients will be randomly assigned to receive either Yijinjing exercise group or brisk walking group or control group in a ratio of 1:1:1. Interventions (Yijinjing exercise or brisk walking training, 40 min per session) will be provided in 3 sessions per week (Monday, Wednesday, Friday) for 12 weeks, with a total of 36 sessions. After the treatment, there will be a 1-month follow-up period. The primary outcomes will be measured using the Montreal Cognitive Assessment (MoCA) and the Unified Parkinson’s Disease Rating Scale motor section (UPDRS-III). Secondary outcomes include balance function, executive function, walking function, sleep quality, and quality of life. Additionally, the prefrontal cerebral and sensorimotor cortex blood oxygen signal level will be collected to explore the underlying mechanisms. All outcomes will be assessed at baseline, at the end of 12 weeks of treatment and after an additional 1-month follow-up period.

**Discussion:**

The results of the study protocol will provide high-quality evidence for the potential of intervention measures based on the Yijinjing exercise to improve the cognitive and activity levels of Parkinson’s disease patients. We envision the Yijinjing exercise as a non-pharmacological family activity that can provide a new and more effective method for the treatment of Parkinson’s disease patients or those at risk.

**Clinical trial registration:**

This study was approved by the Ethics Committee of the Second Rehabilitation Hospital of Shanghai (2020-05-01). The trial has been registered in the China Clinical Trials Registry (ChiCTR2200055636).

## Introduction

Parkinson’s disease (PD) is a chronic progressive neurodegenerative disorder commonly seen in middle-aged and older adults individuals. The disease is characterized by motor symptoms such as bradykinesia, resting tremor, muscular rigidity, postural gait disorders, and non-motor symptoms such as cognitive and emotional disorders, sleep disorders, abnormal defecation, pain, and fatigue (1). PD is considered the second most common neurodegenerative disease globally according to the global burden of disease study (2, 3). In China, the prevalence of PD in people over 65 exceeded 1,700/100,000 from 1990 to 2016 (4), more than doubling the prevalence and exceeding that of any other country (5). Age is the primary risk factor for PD (6), and with the intensification of the aging wave, the disease’s prevalence continues to increase, leading to a significant economic burden on families and negatively impacting the quality of life of the older adults. Therefore, improving the clinical symptoms of PD patients has become a critical public health problem that needs to be addressed.

Currently, there is no practical way to cure PD completely, and drug therapy remains the primary treatment in clinical settings. However, various PD drugs may cause adverse reactions that could affect their therapeutic efficacy (7), which limits patients’ function and decreases their quality of life. While drug therapy cannot prevent or alleviate the process of PD neurodegeneration, it can manage related symptoms of the disease (8). Nonetheless, drug therapy alone is insufficient to meet clinical needs. In recent years, non-drug therapy for PD has gained increasing attention. Previous studies have shown that non-drug therapy can effectively improve the motor symptoms of PD patients and is a crucial strategy for treating non-motor symptoms, such as cognitive impairments that result in disability and reduced quality of life in most PD patients (9, 10). Combining drug and non-drug therapies through a multidisciplinary approach yields benefits beyond drug therapy alone (11).

Active participation in exercise training is a crucial non-drug intervention that can help improve the clinical symptoms of PD patients. Several studies have shown that exercise training can alleviate both motor symptoms (strength, endurance, balance, gait) and non-motor symptoms (cognition, depression, sleep) in PD patients (12–15). Exercise training can also improve cognitive performance and significantly reduce the risk of cognitive impairment or PD in middle-aged individuals, according to relevant laboratory studies (16, 17). However, the optimal type, intensity, duration, and mechanism of exercise training for PD patients still need to be determined. Nonetheless, previous studies have suggested that traditional Chinese exercises (TCEs), as medium-intensity aerobic exercise, can potentially prevent and improve older adults related diseases (18–20).

Yijinjing, an essential part of TCEs, combines movements, breathing and mindfulness, focusing on regulating the balance between body and mind to achieve a healthy state of the body. Moreover, Yijinjing has its unique advantages in TCEs, the 12 movements of the Yijinjing are mostly straight lines, and training requires practitioners to move slowly and coherently. Moreover, during movements training, each movement is relatively independent, and a complete set of movements can be trained, or the movements can be selectively trained according to the practitioner’s own needs. Currently, Yijinjing has demonstrated significant improvements in limb motor function, sleep disorders, cognitive function, and quality of life in older adults and convalescent stroke patients in the community (21–23). Moreover, previous studies have indicated that the Yijinjing has shown benefits in improving the cardiovascular system of the older adults and the motor function of patients with skeletal myopenia (24, 25). Therefore, Yijinjing has played a significant promoting role in improving cognitive and motor functions in the older adults population or patients with neurological diseases. This provides a new treatment method for improving cognitive and motor function in PD patients. However, there is little clinical report on the effectiveness of Yijinjing intervention in PD patients and its potential for treating PD remains to be developed.

Functional near-infrared spectroscopy (fNIRS) is a well-established non-invasive tool to continuously assess regional tissue oxygenation at bed-side. By measuring changes in the light absorption of different hemoglobin species, temporal changes in cerebral blood flow can be calculated with fNIRS. Several lines of evidence have shown that fNIRS is a validated tool for neurocognitive functions (26). Previous studies have suggested that the beneficial role of exercise, may be to facilitate neuroplasticity and improve motor learning. This may occur through enhanced blood flow and alterations in the brain environment (9). Moreover, since prefrontal cognitive circuits are critically involved in early phases of motor learning, another important component of exercise in PD is prefrontal cognitive engagement (9, 27). Therefore, We will observe the changes in cerebral blood oxygen signals between the prefrontal cerebral and sensorimotor cortex in PD during resting and task states using fNIRS.

The main objective of this study is to investigate the impact of a 12-week intervention using Yijinjing exercise on cognitive function, motor function, sleep quality, and quality of life in PD patients. Additionally, this study aims to monitor changes in prefrontal cerebral and sensorimotor cortex blood oxygen using fNIRS and explore possible mechanisms underlying the improvement of cognitive and motor functions in PD patients by this interventions. Based on our hypothesis, Yijinjing exercise could improve the cognitive and motor functions of Parkinson’s patients after 12 weeks of intervention and during a 1-month follow-up, as well as offering benefits for brain network connectivity function.

## Methods

### Study design

**Figure 1:**
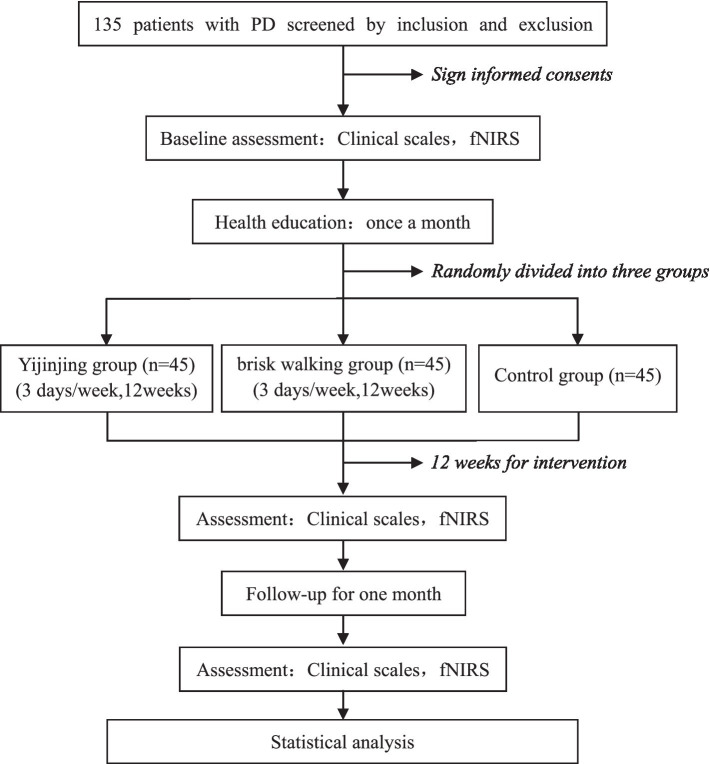
Flow chart of the trial. PD, Parkinson Disease; fNIRS, functional near-infrared spectroscopy; Clinical scales includes: MoCA, Montreal Cognitive Assessment; UPDRS-III, Unified Parkinson’s Disease Rating Scale motor section; BBS, the Berg Balance Scale; TMT, Trail Making Test; 6MWT, 6-MinuteWalk Test; PSQI, Pittsburgh sleep quality index; PDQ-39, Parkinson’s disease questionnaire 39.

This single-center randomized controlled trial will be conducted at the Second Rehabilitation Hospital of Shanghai. Prospective participants will be screened for eligibility and voluntarily signing the informed consent form after invited to participate in the trial. Participants will be randomly assigned to the Yijinjing exercise group, brisk walking group, or control group in a 1:1:1 ratio. All patients will receive routine drug treatment and monthly health education for 12 weeks, while the intervention groups will receive Yijinjing exercise or brisk walking training. The primary outcome measures will be cognitive function and motor function assessed using the Montreal Cognitive Assessment (MoCA) and the Unified Parkinson’s Disease Rating Scale motor section (UPDRS-III), respectively. Secondary outcomes will include balance function assessed using the Berg Balance Scale (BBS), executive function assessed using the Trail Making Test (TMT), walking ability assessed using the 6-min walk test (6MWT), sleep quality assessed using the Pittsburgh Sleep Quality Index (PSQI), and quality of life assessed using the Parkinson’s Disease Questionnaire 39 (PDQ-39). Exploratory outcomes will include changes in cerebral blood oxygen signals in the prefrontal cerebral and sensorimotor cortex. Outcome measures will be collected before and after the interventions and after 1 month of follow-up. The assessor and data analyst will be blinded throughout the trial. The procedure is illustrated in Figure 1 and Table 1.

**Table 1:** Schedule of enrolment, intervention, and assessments.

Study period					
Time point	Enrolment	Baseline	Postallocation		Follow-up
	−1 week	0 week	1 week	12 week	1 month
**Enrolment**					
Eligibility screen	**×**				
Informed consent	**×**				
Descriptive information^*^		**×**			
Randomization		**×**			
Allocation		**×**			
**Intervention**					
Control group			**×**	**×**	
brisk walking group			**×**	**×**	
Yijinjing group			**×**	**×**	
**Assessments**					
MoCA^*^		**×**		**×**	**×**
UPDRS-III^*^		**×**		**×**	**×**
BBS^*^		**×**		**×**	**×**
TMT^*^		**×**		**×**	**×**
6MWT^*^		**×**		**×**	**×**
PSQT^*^		**×**		**×**	**×**
PDQ-39^*^		**×**		**×**	**×**
fNIRS^*^		**×**		**×**	**×**
Adverse events		**×**	**×**	**×**	**×**

^*^Descriptive information includes the personal general data, vital signs, history of present illness, clinical symptoms and signs, medical history, medication history and neurological examination. “**×**” means the item will be completed. MoCA, Montreal Cognitive Assessment; UPDRS-III, Unified Parkinson’s Disease Rating Scale motor section; BBS, the Berg Balance Scale; TMT, Trail Making Test; 6MWT, 6-MinuteWalk Test; PSQI, Pittsburgh sleep quality index; PDQ-39, Parkinson’s disease questionnaire 39; fNIRS, functional near-infrared spectroscopy.

### Sample size calculation

The design of this study is novel. According to the trial design and previous literature assessment, the sample size will be calculated based on hypothetical changes in MoCA score (28) or UPDRS motor score (10) at week 12. With a power of 90% and an alpha of 5% (two-sided). The SD from our feasibility study is used and our power calculation considered our planned analysis method, by means of an analysis of covariance (ANCOVA) in which baseline measurements served as a covariates, reducing the needed sample size (29). we calculated the minimum sample size (effect size = 0.3) in MoCA score (SD = 2.07, Difference = 1.8) to be 28 participants per group and UPDRS motor score (SD = 2.55, Difference = 1.9) to be 38 through PASS software (version 15.0.5). Therefore, With a 15% dropout rate considered, a total sample size of 135 participants will be necessary.

### Participants

#### Inclusion criteria

Participants must meet the following conditions: (1) the diagnostic criteria of Parkinson’s Disease in China (2016) (5), Hoehn Yahr grade of I–III; (2) presence of mild cognitive impairment (MCI), not demented: MoCA score between 21 and 26 points (30); (3) aged between 60 and 75 years; (4) stable disease at least 3 months before randomization (confirmed to have no acute aggravation and related drug changes); (5) can cooperate and complete the relevant evaluations and tests; and (6) willing to provide written informed consent.

#### Exclusion criteria

Exclusion criteria are as follows: (1) patients with severe bone, joint, and muscle diseases, as well as heart, liver, kidney, and other serious life-threatening diseases or mental illness; (2) patients with severe language, visual, and hearing impairment; (3) patients who have suffered from other severe diseases in the past year which may affect motor ability or cognitive function; (4) patients who have experience doing TCEs in the past; and (5) patients who are participating in other clinical studies.

### Randomization and blinding

Participants will be assigned randomly to one of the three groups using a random number table generated by SPSS V.24.0 to minimize bias. A designated researcher who will not be involved in any other aspect of the study will be responsible for implementing the randomization scheme. Random numbers will be placed in opaque envelopes, and only the designated researcher will know the assigned sequence. The therapists will know the treatment plan for each group, the evaluators and data analysts will be blinded until statistical analyses are completed. Participants will only be informed of their treatment methods in their respective groups and will not be aware of the treatment plans of other participants. All researchers will undergo training before the trial to successfully implement randomization and blinding.

### Baseline assessment

Before the outset of the study, we will gather comprehensive descriptive information from all participants, including general data (name, gender, age, etc.), clinical symptoms (symptom screening for PD and MCI), vital signs (pulse, breathing, blood pressure, etc.). This information will allow us to compare the baseline characteristics of the three groups involved in the study. To further supplement this data, we will conduct the first clinical-scale evaluation and acquire cerebral blood oxygen signals from the prefrontal cerebral and sensorimotor cortex during the baseline assessment. Together, this data will provide valuable insights into the study’s starting point and help us draw more accurate conclusions from the research.

### Intervention

All participants will receive monthly health education as part of the intervention. Each session will be 60 min, covering topics such as the concept, risk factors, and self-management of PD. The intervention will be conducted by licensed therapists with at least 5 years of experience. The control group will be instructed to maintain their existing daily habits. The Yijinjing exercise group will be trained by an expert in TCEs and receive guidance throughout the 12-week program, while the brisk walking group will receive treatment from a professional technician. The intervention will be conducted once a day, 3 days a week (Monday, Wednesday, and Friday) for 12 weeks. Participants will be allowed to continue their current drug treatment as long as it benefits their condition.

### Yijinjing exercise group

Participants in this group will engage in a 12-week Yijinjing exercise program consisting of 12 movements. These movements adhere to the standards established by the General Administration of Sport of China^1^ and will be practiced alongside background music. A picture of the specific movements is available in a previously published article (31). Each exercise session will last for 40 min, divided into 5 min of muscle stretching as preparation, 30 min of Yijinjing exercise, and 5 min of free walking to cool down. The same therapist will supervise the entire exercise process for all participants. Participants who cannot complete the movements independently can receive assistance from the therapist.

### Brisk walking group

Participants in this group will receive brisk walking training. Adopting a moderate intensity aerobic exercise method, with a dedicated therapist supervising the entire exercise process for all participants. 40 min each exercise session, consisting of a 5 min warm-up, 30 min of walking training and a 5 min cool-down. The heart rate during walking should be 50%–60% maximum heart rate (220—age). Monitored by sport watches in heart rate, distance, speed, and walking quality (32).

### Follow-up

After 12 weeks of treatment, participants will undergo a 1-month follow-up period. As they will not have access to equipment and professionals during this period, we will not require them to continue with the corresponding treatment but instead encourage them to resume their daily routine. After the 1-month follow-up, all participants will be required to return to the hospital to reassess of the results.

### Outcome measurements

This research will assess the primary and secondary outcomes and the cerebral blood oxygen signal level by collecting data at baseline, after 12 weeks of treatment, and again at the end of a 1-month follow-up period. To ensure impartiality, all outcome assessments will be conducted by an experienced therapist who is unaware of the randomization of the participants.

#### Primary outcome

Cognitive and motor functions will be measured using the MoCA and UPDRS assessments, respectively. The MoCA evaluates 8 aspects of the cognitive domain, including visual space and execution, naming, memory, attention, language, abstraction, delayed recall, and orientation (33). With a total score is 30, a score of ≥26 indicates no cognitive impairment. Alternate Chinese versions 7.1 and 7.2 will be used to minimize the learning effect before and after the treatment. On the other hand, the UPDRS is the most widely used, well validated clinical rating scale for PD, UPDRS-III is the motor section of UPDRS, through 18 tasks, each scored from 0 to 4, with a maximum score of 72 (34).

#### Secondary outcomes

Secondary outcomes of this study include balance function, executive function in specific cognitive areas, walking function, sleep, and quality of life.

##### Balance function

Berg Balance Scale (BBS) measures static and dynamic balance abilities through 14 tasks, each scored from 0 to 4, with a maximum score of 56 (35).

##### Executive function

Executive function will be assessed using the Trail Making Test (TMT) (36), which consists of two parts, A and B. Part A requires participants to connect irregularly scattered numbers from 1 to 25 as quickly and accurately as possible. Part B includes numbers and letters, which are required to be connected in the order in which numbers and letters cross, such as 1 connected to A, A connected to 2, 2 connected to B, etc. The time it takes to complete and the number of errors across both parts will be recorded.

##### Walking function

The walking function will be assessed using the 6-Minute Walk Test (6MWT) (37), which requires participants to walk back and forth as much as they can over the course of 6 min. Participants are not allowed to talk while walking or run and jump. Researchers will follow participants to protect them and record their total walking distance.

##### Sleep

Sleep will be assessed using the Pittsburgh Sleep Quality Index (PSQI) (38). Participants will complete a self-assessment, including 7 parts consisting of 18 self-assessment items, with scores ranging from 0 to 21. In general, the higher the score, the worse the sleep quality.

##### Quality of life

The Parkinson’s Disease Questionnaire 39 (PDQ-39) will assess the quality of life (39). PDQ-39 consists of 39 items, assessing 8 dimensions of physical activity, daily life behavior, mental health, humiliation, social support, cognition, communication, and physical discomfort. The score ranges from 0 to 156. The higher the score, the lower the quality of life.

#### Exploratory outcomes

**Figure 2:**
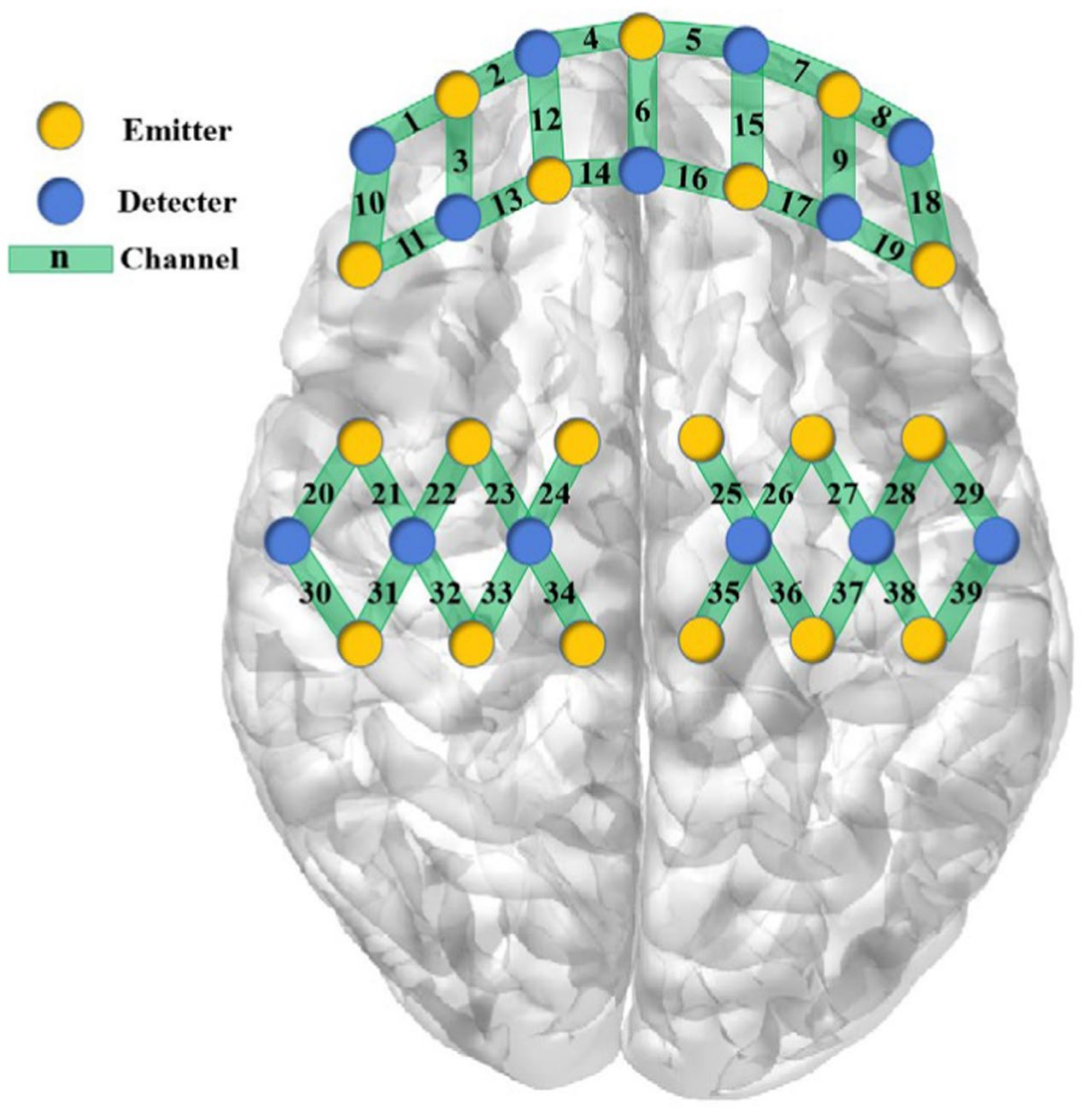
Configuration of fNIRS channels.

The near-infrared brain functional imaging system (NirSmartII-3000A, Danyang HuiChuang Medical Equipment Co., Ltd., Danyang, China) will be operated by trained professionals to capture cerebral blood oxygen signals in the prefrontal cerebral and sensorimotor cortex. The device features 16 emission and 24 detector probes, with 64 channels available. To ensure consistency, participants will wear an A-type optical fiber cap configured by the instrument and their prefrontal and sensorimotor region will be located according to the 10–20 system marker points. Shown in Figure 2. To elicit task-based responses, participants will be asked to perform three tasks.

**Figure 3:**
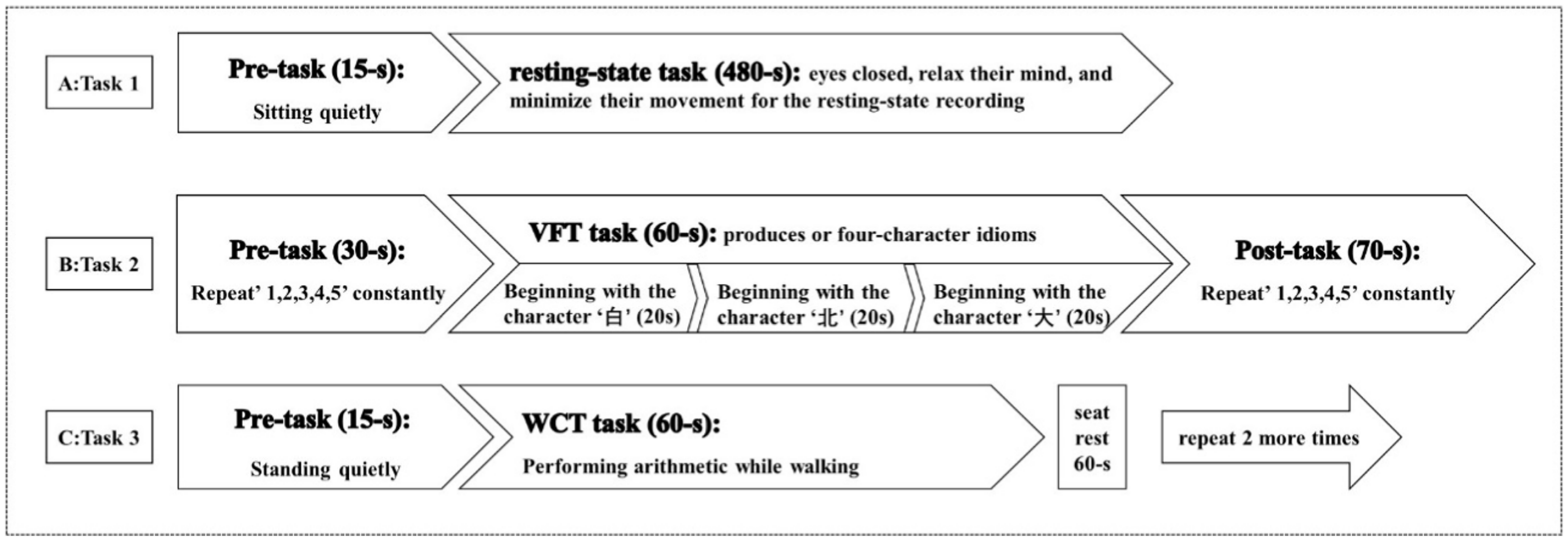
Three tasks for fNIRS.

**Task 1:** Participants wore the fNIRS detection cap and sat in a chair. They were instructed to keep still with their eyes closed, relax their mind, and minimize their movement for at least 8 min for the resting-state recording (40) (Figure 3).
**Task 2:** the verbal fluency task (VFT): Participants wore the fNIRS detection cap and sat in a chair. The task time is about 160 s. The first 30s and the last 70s of the experiment are used to collect the resting-state signal of the participants. During these two stages, the participants are required to count from one to five repeatedly. During the second stage, the participants performed VFT of three consecutive word-generating tasks, each of which lasted 20 s (41) (Figure 3).
**Task 3:** Walking while performing cognitive task (WCT): Participants will be asked to walk on a 30-meter walkway. They will be first provided with 2 familiarization trials of the walking condition to become accustomed to walking with the fNIRS backpack. The task time is 75 s. Participants stand quietly at the beginning of the corridor for the first 15 s of the experiment, and then walk back and forth at a comfortable walking speed along a straight distance of 30 meters for the last 60 s while subtracting 3 from an initial three-digit number (between 900 and 1,000) serially and speaking out each calculated number as quickly as possible. Repeat 3 times, with a 60 s seat rest between each session (Figure 3).

The fNIRS data will be analyzed using the Homer2 package in MATLAB and customized MATLAB-based script (42).

##### Safety

All adverse events that occur during the trial will be documented, regardless of whether they are related to the study. The ethics committee will be promptly notified if any severe adverse events arise. A clinician from the Department of Neurology of the Second Rehabilitation Hospital of Shanghai will provide the necessary treatment. The ethics committee will evaluate the correlation between the adverse events and the trial intervention and will determine whether the participant can continue the trial, which will be accurately recorded in the case report form (CRF).

##### Quality control

The trial study will establish a unique quality control group in order to train participants in recruitment, assessment, treatment, and data analysis so as to minimize bias. Throughout the trial, the quality control group will strictly record medication taken by participants and be responsible for communicating with participants and monthly health education. The intervention group will each assign a designated therapist to carry out one-on-one free treatment throughout the trial to reduce the differences in therapeutic effects between therapists. The above measures will assist in improving participant compliance and reduce the loss rate of follow-up.

##### Data management and monitoring

To safeguard participants’ privacy, the evaluator will assign an identification code to each individual, concealing their personal information. Data collection will be performed using paper CRFs, which will be handed over to an authorized research assistant on the same day as each completed evaluation. The data collected will be converted into electronic format and securely stored on an encrypted hard drive.

### Statistical analysis

All primary and secondary analyses will be conducted on an intention-to-treat (ITT) basis. Modified ITT analysis included all randomized participants who completed at least 8 weeks of the intervention. Primary analyses will be conducted using multiple imputation methods for missing observations at baseline, post intervention, and at follow-up, assuming missing data are missing at random. Prespecified sensitivity analyses will be conducted for all participants using complete-case data at each time point.

Continuous data will be presented as mean ± SD or median ± quartile interval, while enumeration data will be presented as numbers and percentages. Between-group differences in demographic and baseline variables, as well as assessment variables, will be assessed using a chi-square test or the Fisher exact test for categorical variables and a one-way analysis of variance (ANOVA) or the Kruskal-Wallis test for continuous variables as appropriate. Intervention effects on primary and secondary continuous outcome measures will be compared by means of mixed repeated-measures analysis of variance, with and without adjustment for baseline and time-varying covariates (e.g., age, sex, and Hoehn and Yahr stage). Pairwise comparisons between the Yijinjing group and the two other groups will be conducted only if the omnibus F-test statistics indicated that the null hypothesis should be rejected. The primary analysis will to determine whether Yijinjing training improved the MoCA and UPDRS-III score compared with brisk walking training and control at 12 weeks. These continuous outcomes will be assessed by the 1-way analysis of variance model at 12 and 16 weeks. All statistical analyses will be conducted using SPSS software, version 24 (IBM). *p* value < 0.05 will be considered statistically significant. An independent statistician will carry out all statistical analyses.

## Discussion

Yijinjing is a type of TCE that requires practitioners to synchronize their thoughts, breathing, and movements in all directions of the body. The practice aims to achieve a harmonious balance between mind and body and coordinate the entire body’s functions. Our previous studies found that older adults in the community demonstrated a high level of interest and acceptance in practicing Yijinjing exercises. Our research also confirmed that Yijinjing training significantly enhances cognitive function and sleep quality in older adults and stroke patients (21, 22). Yijinjing can also improve balance function and gait in stroke patients, further highlighting the potential of this practice to improve both cognitive and motor functions (43).

However, most previous studies have focused on improving motor symptoms in PD patients, with relatively little attention paid to non-motor symptoms. Among the most common non-motor symptoms in PD, cognitive impairment is a significant concern, which can be divided into two categories: mild cognitive impairment in PD (PD-MCI) and dementia in PD (PD-D). Pigott et al. reported that approximately 50% of PD patients with normal cognitive function will develop cognitive impairment within 6 years of onset (44). Without timely intervention, 70%–80% PD-MCI will develop into PD-D (45). According to existing studies, the pathological mechanism of cognitive impairment in PD is unclear, but it may be related to α-synuclein, β-amyloid deposition, neurotransmitters, and other factors (46). Functional brain imaging studies have shown that cognitive and motor functions share parts of the brain network, particularly in the prefrontal regions (47–50). PD cognitive impairment is characterized by functional abnormalities in many areas, including attention, memory, language, visual space, and executive function. The prefrontal lobe plays a vital role in executive function, which involves continuous and selective attention, response inhibition, and working memory (51). Given that executive function, attention, and memory are closely related to motor function, it is believed that cognitive impairment in PD patients may affect the rehabilitation of their motor symptoms and reduce their quality of life. Therefore, solely focusing on improving motor symptoms in PD patients is insufficient. The management of cognitive function in PD patients should be strengthened, and efforts to improve motor function should be promoted simultaneously.

Therefore, this study aims to observe the effectiveness of Yijinjing exercise in improving the cognitive and motor functions of PD patients. The related mechanisms will be explored by analyzing cerebral blood oxygen signal levels in the prefrontal cerebral and sensorimotor cortex, as well as the interaction between cognitive and motor function in PD patients.

## Ethics statement

The studies involving humans were approved by the Ethics Committee at the Second Rehabilitation Hospital of Shanghai. The studies were conducted in accordance with the local legislation and institutional requirements. Written informed consent for participation in this study was provided by the participants’ legal guardians/next of kin.

## Author contributions

KL: Writing – original draft, Writing – review & editing. XM: Writing – original draft, Writing – review & editing. XJ: Project administration, Writing – original draft. XL: Project administration, Writing – original draft. YL: Project administration, Writing – original draft. SM: Writing – original draft, Writing – review & editing. JH: Writing – original draft, Writing – review & editing.

## Footnotes

^1^ http://www.Sport.gov.cn/
